# Is It Possible to Forgive Child Sexual Abuse?

**DOI:** 10.3389/fpsyg.2020.619597

**Published:** 2021-01-21

**Authors:** María Prieto-Ursúa

**Affiliations:** Department of Psychology, Universidad Pontificia Comillas de Madrid, Madrid, Spain

**Keywords:** child sexual abuse, forgiveness, justice, reconciliation, unconditional forgiveness

## Introduction

Child sexual abuse has been termed “the absolute evil” (Tener and Eisikovits, 2015), and its negative consequences, in the short and long term, have been widely documented. In addition to numerous psychopathological problems, the consequences for the victims' personal, emotional, and behavioral experience have been described thus (Browne and Finkelhor, 1986): traumatic sexualization, stigmatization, helplessness, and if the abuser was trusted, the feeling of betrayal. Furthermore, when the abuser is a clergyman, “spiritual devastation” or “the death of the soul” has also been described (Benkert and Doyle, 2009). If there were any unforgivable behavior, it would probably be this.

Even merely raising the possibility of forgiveness in child sexual abuse is delicate in itself. At times, forgiveness has been used by abusers as a means of guaranteeing the victim's silence (Casey, 1998). The pressure by the community on the victim to forgive can be understood as contempt for his or her suffering, as a way of detracting from the seriousness or importance of the abuser's behavior (Rudolfsson and Tidefors, 2015) or as a way of releasing him from responsibility or from deserved punishment (Tracy, 1999), making revictimization more likely (Tener and Eisikovits, 2015). According to Casey (1998), any therapeutic approach that insists on the need for forgiveness has fallen time and time again into the trap of denying the child the space to show the pain of their childhood; it is an attempt to close the wound before cleaning or healing it.

However, these consequences appear when erroneous concepts of forgiveness are proposed, concepts that confuse forgiveness with absolution, with reconciliation or with behaving as if “nothing has happened here.” These ways of understanding forgiveness do not free one from abuse; they only perpetuate it and facilitate future disrespect and a lack of consideration toward the victim. Prolonged abuse in a relationship creates complex, coercive interactions between the victim and the abuser, combining violence, and dependency (Lahav et al., 2019); misconceptions of forgiveness can further weaken a victim's ability to protect themselves, thereby making them more vulnerable, and facilitating the continuation of the abuse. You cannot truly forgive if you cannot freely forgive (Tracy, 1999), and this does not happen until the circle of victimization and helplessness in which the victim lived has been broken, and then they can move from victim to survivor.

Forgiveness is a complex concept, with multiple dimensions and possibilities, and it can offer victims a valuable resource for overcoming their pain. Although forgiveness is not essential to a victim's healing process, there are numerous studies that have discussed the positive effects of forgiveness in victims of sexual abuse (Freedman and Enright, 1996; Kaminer et al., 2000; Lee and Enright, 2014).

First, forgiveness must be clearly differentiated from reconciliation. Forgiveness is an individual process, a change in the heart of the victim which leads to a reduction in the discomfort experienced when one is the victim of a serious offense, thus helping to mitigate and alleviate negative emotions (anger, hostility, resentment, hatred, anger, shame, humiliation) and negative thoughts (repetitive and intrusive thoughts about the offender, the situation, or the action itself) and reducing the tendency to show avoidance or revenge behavior (McCullough, 2008). Reconciliation is a two-part process that aims to restore relationships and trust. Not all forgiveness processes involve reconciliation. If we do not differentiate the two processes well when we consider the possibility of forgiveness, the victim may renounce forgiving, thinking that it implies relating again to the abuser (Freedman, 1998). Helm et al. (2005) showed that most of the victims of abuse they interviewed indicated that they preferred to keep their distance from the abuser, regardless of whether they said they had forgiven him or not.

Forgiveness without reconciliation arises in situations in which there is no guarantee that the offense will not be repeated or in situations in which the relationship is not equal and that true reconciliation is therefore impossible. Resuming the relationship with the abuser could only be considered, according to some authors (Cooney et al., 2011), when there are indicators of genuine repentance on the part of the offender: assuming full responsibility for the abuse (confessing), recognizing the magnitude of the damage caused to the victim, showing remorse for causing it, showing respect for the victim by setting limits so that the abuse does not reoccur, and taking steps to change the disruptive behavior patterns that led to the abuse.

Second, forgiveness should not be confused with the absence of the need for justice. True forgiveness does not interrupt the process of justice nor eliminate the punishment the abuser deserves for his conduct. You can forgive and still seek justice. Let us remember that forgiveness happens inside the victim and frees him or her from post-offense hate and suffering. The trial and conviction processes of the abuser for his conduct are independent of this process. An unbalanced strategy of seeking compassion could suppose a kind of forgiveness that ignores justice (Enright et al., 1992).

Third, forgiveness can be understood as an exclusively individual process, which the offended person goes through unconditionally, unidirectionally, without the participation of the offender, or it can be understood as a two-person process, in which conditions must be established for the abuser so that forgiveness is possible (normally, the admission of responsibility, a demonstration of repentance, and some reparative behavior). This negotiated forgiveness has been advocated as the appropriate forgiveness for victims of child sexual abuse (Casey, 1998). For this author, forgiveness is not unidirectional, unconditional, or individual, but rather occurs in an interpersonal context and requires conditions.

However, if forgiveness depends on the abuser's behavior, the abuser maintains his power over the victim, deciding whether or not the victim can begin a forgiveness process and decreasing her capacity of control (Tracy, 1999). Thus, processes have been proposed to facilitate forgiveness among victims of child sexual abuse based on individual, unconditional forgiveness (Tracy, 1999; Walton, 2005). These proposals follow the steps suggested in the main interventions to facilitate forgiveness (Enright and Fitzgibbons, 2000) and adapt them to the particular characteristics of this offense, sexual abuse. They propose a deep and liberating work after which the victim comes out strengthened and recovers his or her sense of control, a key element in the healing process for victims of sexual abuse.

Finally, I would like to point out some essential elements to raise the work on forgiveness with victims of abuse. Specifically, it is suggested that the following recommendations be considered. (1) Remember that forgiveness is a difficult, long, slow process, so it is advisable to take the necessary time and not rush processes. (2) It is important to validate the feelings that the victims express without blaming them, offering support. (3) At some point, the victims must accept that the offense has occurred and that it is part of their own lives; it is not about trying to act “as if nothing ever happened” and forgetting it but about finding a place for it and being able to continue living. The recovery process includes finding hope in the future and meaning in life (Morton et al., 2019). (4) It is important to overcome helplessness and lack of control and to facilitate responsibility for one's life, for the future. (5) It is essential for the victims to set boundaries, to decide on the people they want in their lives and how they want to be treated by those people. (6) Self-forgiveness is a fundamental step in the recovery of victims; it helps the victims understand that they are not the problem, but that the problem is the grossly incorrect and unfair behavior of the aggressor and the confusion generated. (7) If the victims say that they may consider the possibility of reconciliation, inquire whether they do so realistically, with adjusted expectations about the offender (Tener and Eisikovits, 2015). (8) Finally, beware of social pressure (Tener and Eisikovits, 2015), the existence of inflexible norms, and the expectations of victims that may have been communicated to them, e.g., that the abuse is unforgivable, that they must forget the abuse and not talk about it, that they must forgive in order to protect the integrity of the family or the community, or that they must be “the eternal victim” and never forgive in order to make the seriousness of the offense clear.

## Conclusion

Forgiveness can be proposed as a tool to help and heal the pain and suffering of victims of child sexual abuse, but always being very careful when offering it, avoiding transmitting to victims any kind of moral obligation and maintaining a concept of forgiveness that respects justice and protects them.

## Author Contributions

The author confirms being the sole contributor of this work and has approved it for publication.

## Conflict of Interest

The author declares that the research was conducted in the absence of any commercial or financial relationships that could be construed as a potential conflict of interest.
